# Cd Stress Response in Emmer Wheat (*Triticum dicoccum* Schrank) Varieties Under In Vitro Conditions and Remedial Effect of CaO Nanoparticles

**DOI:** 10.3390/biology14040394

**Published:** 2025-04-09

**Authors:** Doğan İlhan, Büşra Yazıcılar

**Affiliations:** 1 Department of Molecular Biology and Genetics, Faculty of Science and Letters, Kafkas University, Kars 36000, Türkiye; 2 Department of Molecular Biology and Genetics, Science Faculty, Erzurum Technical University, Erzurum 25050, Türkiye; busra.yazicilar21@erzurum.edu.tr

**Keywords:** Kavilca wheat, *5BL*, *6BL*, CdCl_2_, CaO NP, in vitro

## Abstract

In this study, the mitigating effects of CaO NPs obtained from pomegranate extract via environmentally friendly green synthesis on CdCl_2_ stress in two varieties (*Yolboyu* and *Kirac*) of Turkish Kavilca wheat (*Triticum dicoccum* Schrank) under in vitro callus culture conditions were investigated. The calluses developed from embryos of both wheat varieties were exposed to either CaO NPs alone (1 and 2 mg/L), CdCl_2_ alone (1 or 10 mM) or the different combinations of these two compounds in MS medium for 4 weeks. Changes in the expressions of two genes (*Traes_5BL_9A790E8CF* and *Traes_6BL_986D595B9*) known to be involved in wheat’s response to CdCl_2_ stress were analyzed by qRT-PCR. Additionally, certain physiological parameters, such as lipid peroxidation (LPO), H_2_O_2_, proline and soluble sugar content, and SEM-EDX analysis were used to assess the response of calluses to the applications. The CaO NPs treatments alone generally upregulated the expression of the *5BL* and *6BL* genes, while the CdCl_2_ applications decreased their expression in both cultivars. The CaO NPs reduced the proline content in both cultivars compared to the control. Co-treatment with CdCl_2_ and CaO NPs increased the sugar content and decreased the MDA content, but did not cause a significant change in the H_2_O_2_ content. SEM analysis showed that when CdCl_2_ and CaO NPs were applied to calluses together, the membranous and mucilaginous spherical structures were regained. The application of CaO NPs reduces the amount of cellular damage caused by CdCl_2_ stress and improves gene expressions.

## Simple Summary

Cadmium (Cd) toxicity, a significant global issue, directly impacts agricultural ecosystems, leading to yield losses in cereal crops like wheat. Cd also disrupts the uptake and utilization of the essential nutrient calcium (Ca) from the soil. Minimizing Cd absorption in cereals is therefore critical, but current strategies are limited. One promising approach involves using calcium oxide nanoparticles (CaO NPs), an innovative tool that could aid in reducing Cd uptake. In this context, ancestral emmer wheat, a species-rich in genetic diversity and cultivated on a limited scale in Türkiye, has gained attention. Genotypes of this species may provide effective solutions for reducing Cd absorption, transport, and their associated negative effects. Additionally, studying variations in local genotypes may help clarify the molecular and physiological mechanisms underlying these processes. This study aimed to investigate the effects of different levels of Cd and CaO NPs on emmer wheat varieties grown in vitro. By examining gene expression and physiological parameters linked to Cd stress, it was determined that CaO NPs serve as a useful strategy for mitigating cellular damage caused by Cd toxicity.

## 1. Introduction

Cadmium (Cd), a highly toxic heavy metal, poses a significant threat to agricultural ecosystems worldwide, particularly in regions where soil contamination is prevalent. Agricultural soil typically has a regulatory limit of 100 mg/kg for Cd content. With its increasing presence in farm soils, Cd endangers crop productivity and raises concerns about food safety. Many agricultural areas are contaminated with Cd due to industrial waste, especially from phosphate fertilization activities and other polluting wastes [1,2,3,4]. This contamination leads to high levels of Cd accumulation in cereals such as wheat, thereby significantly reducing yields due to its toxic characteristics and non-essential properties [5]. Additionally, Cd interferes with various physiological processes in plants, such as inhibiting root Fe (III) reductase activity, leading to iron deficiency and affecting photosynthesis [6].

Cd also interferes with the uptake, transport and utilization of essential elements like calcium, magnesium, phosphorus and potassium. Cd toxicity alters plasma membrane permeability, reduces water content and inhibits ATPase activity in wheat and sunflower roots [1]. Calcium (Ca), however, is the main macronutrient that acts as a growth and development regulator in wheat and other plants [7]. Toxic factors such as osmotic changes and heavy metals in plant cells change the Ca^2+^ ratio in plant cells [8]. Cd’s structure resembles the structure of Ca^2+^ and, therefore, Cd can be transported and absorbed by Ca^2+^ channels in plants [9]. Competition between the movements of Cd and Ca in plants are quite common [10]. Under Cd stress conditions, oxidative stress occurs due to excessive production of Reactive Oxygen Species (ROS). Increased oxidative stress in plants disrupts several processes, compromising cellular integrity, damaging DNA and altering protein structures. Consequently, oxidative stress impairs growth, development, and overall physiological functions in plants [1,9]. One common outcome of Cd exposure is high levels of lipid peroxidation, typically resulting in the production of end products like malondialdehyde (MDA) [11].

In addition, various gene families, such as pathogenesis-related proteins and aldehyde dehydrogenases, play a crucial role in the response to Cd stress at the cellular level. *Traes_5BL_9A790E8CF* and *Traes_6BL_986D595B9* are two important gene groups that change their expression levels under Cd stress, making them particularly important in determining the unfavorable effects of Cd heavy metal in winter wheat (*T. aestivum*) species [12,13]. These genes are thought to be useful for the discovery of associated genes involved in transcription, metal transport, and signal transduction processes. Furthermore, pathogenesis-related proteins can also be induced by Cd stress [14]. To the best of our knowledge, no study has investigated the response of genes in *T. dicoccum* (emmer wheat) to Cd stress.

Minimizing the uptake of Cd from the soil by cereals is a vital global challenge, but strategies aimed towards this purpose are limited [15]. Nanotechnology provides innovative tools that can play a critical role in addressing the issue of heavy metal toxicity in agriculture [16]. Metallic nanoparticles (NPs) are increasingly favored for their efficacy in remedying the adverse effects of heavy metal contamination [17]. Characterized by their colloidal nature and minute size, typically under 100 nm, NPs offer unique advantages in environmental remediation [18,19]. The application of nanoparticles in plants is critical in reducing cadmium (Cd) toxicity. Nanoparticles such as titanium dioxide (TiO_2_) and zinc oxide (ZnO) have been shown to reduce oxidative damage, and thus alleviate Cd stress [20,21]. For example, TiO_2_ nanoparticles increase seed viability in lentils exposed to Cd and provide support by reducing lipid peroxidation [20]. In addition, calcium disodium EDTA nanoparticles have been reported to effectively chelate cadmium, minimizing its toxic effects on plant tissues [22]. Studies also show that nanoparticles can provide plants with resistance to cadmium stress by increasing the bioavailability of essential nutrients [23]. Nowadays, the development of useful and practical green technology for metal NP recovery is promising. In this context, one innovative method is obtaining metal nanoparticles from plants [24]. Beyond traditional methods, green synthesis methodologies using nanotechnology demonstrate a high efficiency in minimizing the effects of harmful synthetic compounds, emphasizing their importance as a key component of environmentally friendly procedures. An important nanomaterial produced by this method is calcium oxide (CaO) NPs. These particles are now widely utilized for different purposes today, such as agricultural applications, plant growth and development analyses, the determination of germination capacity, monitoring of leaf development, toxicity and tolerance tests [25] and the determination of stress-induced physiological responses [26].

The problem of cadmium contamination in wheat farming in Türkiye is an urgent concern due to its potential effects on both agricultural yield and human health. Cadmium accumulation in wheat is due to various factors such as soil properties, agricultural practices, industrial wastes and irrigation methods [27,28]. The tetraploid wheat *T. dicoccum* (emmer wheat) is still grown in limited quantities by some farmers in the northwest and northeast Anatolian region (Türkiye). It is also known that Gernik, Kavilca, and Kabulca, grown in the Kars province and its surroundings, are the wild ancestors of grasses and have a history of approximately 13 thousand years [29]. A low gluten protein content has been detected in the flour obtained from Kavilca wheat, and due to this feature, this wheat has been preferred by celiac and cancer patients as it can be easily digested [30]. Xiao et al., (2019) [14] suggest that such a genotype may influence Cd uptake, accumulation, translocation and detoxification. However, the potential molecular mechanisms governing these processes remain unclear and could be better understood by examining local genotype variations. Additionally, employing these genotypes can present an efficient and practical approach to mitigating dietary Cd exposure [14,31].

The objective of this study was to investigate the impact of CaO NPs, combined with varying concentrations of CdCl_2_, on two Kavilca wheat cultivars (*Triticum dicoccum* Schrank, cvs. *Yolboyu* and *Kirac*) under in vitro callus culture conditions. As such, our primary objective was to evaluate the expression profiles of pertinent reference genes, specifically focusing on genes located on chromosomes *5BL* and *6BL*. Concurrently, we aimed to analyze key physiological indicators encompassing malondialdehyde (MDA) levels, hydrogen peroxide (H_2_O_2_) concentrations and proline and soluble sugar levels. Also, we intended to conduct Scanning Electron Microscopy–Energy Dispersive X-ray Spectroscopy (SEM-EDX) examinations to determine the extent of tissue damage. This comprehensive approach was designed to furnish a detailed understanding of the cellular and molecular responses to the imposed stressors, thereby contributing to a nuanced comprehension of the underlying mechanisms involved.

## 2. Materials and Methods

### 2.1. Plant Material

In the present study, *Yolboyu* and *Kirac* Kavilca wheat (*Triticum dicoccum*) cultivars were used as the plant material. The plants’ seeds were ancestral seeds obtained from local farmers in the Kars province (Türkiye). The decontamination of the seeds’ surfaces was carried out by keeping them in 70% ethanol for 10 min, in 10% NaOCl and Tween 20 for 30 min and washing them 3 times with sterile dH_2_O. The seeds were incubated overnight at 4 °C.

### 2.2. Tissue Culture and Callus Formation

MS medium [32] was prepared and autoclaved under 1 atm pressure at 121 °C for 15 min. After autoclaving, a solution of 2 mg/mL 2,4-Dichlorophenoxyacetic acid (2,4-D) hormone was filtered through a 0.25 micrometer sterile filter and then mixed into MS medium. The prepared mixture was poured into a sterile Petri dish with a diameter of 9 × 9 mm. *Yolboyu* and *Kirac* embryos were seeded in the MS medium. Embryos were grown in the Petri dishes with MS at 25 ± 1 °C and 1500 lx lighting conditions for 4 weeks under a 16/8 h long day photoperiod. Mature embryos were solidified in modified MS medium under aseptic conditions with 2 mg L^−1^ glycine, 0.5 mg L^−1^ Myo-inositol, 0.1 mg L^−1^ pyridoxine, 0.5 mg L^−1^ thiamine, 0.5 mg L^−1^ nicotinic acid, 1.95 g MES, 20 g sucrose and 7 g agar. Autoclaving was performed and the pH was adjusted to 5.8. A 0.22 µm porous cellulose nitrate filter was utilized during the sterilization process with 50 mg L^−1^ ascorbic acid and 4 mg L^−1^ 2.4-D. Mature embryos were incubated at 25 ± 1 °C in complete darkness for 4 weeks.

### 2.3. Green Synthesis and Characterization of CaO NPs

The pulp of a pomegranate fruit (*Punica granatum* L.), sourced from the Department of Biology, Science Faculty, Atatürk University (Türkiye), was utilized in the preparation of the plant extract for synthesizing CaO nanoparticles. For this purpose, first, the pomegranate peels were removed and the seeds were removed, and then 25 g of pomegranate seeds were crushed in 100 mL of distilled water with the help of a blender. After filtration with a filter paper, centrifugation was performed at 5000× *g* for 10 min. The liquid fraction was removed after centrifugation to use in the green synthesis process [33].

Production of CaO NPs was performed using 0.1 mM CaCl_2_ and the supernatant extract obtained from pomegranate fruit [34]. For green synthesis, first of all, the pomegranate extract was obtained into 10 mL, and 100 mL of 0.1 mM CaCl_2_ was added in a stirrer at 300 rpm and refluxed at 800 °C. Samples from the reaction were analyzed in a spectrophotometer for 72 h. It was found that CaO NPs reached maximum absorbance at 427 nm in the spectrum between wavelengths of 190 and 1000 nm. In this process, optimizations of basic parameters such as time, pH and ion concentration were also provided separately. CaO NPs obtained from this process, called green synthesis, were washed with distilled water–ethyl alcohol and dried in an oven at 40 °C overnight [32]. Characterization of the nanoparticles was achieved by verifying their size, structure, and surface properties using SEM, XRD and FTIR analyses [34].

### 2.4. CdCl_2_ Stress and CaO NP Application

For cadmium stress and CaO NPs treatments, mature embryos of the cultivars were exposed to MS nutrient medium containing 1 mg/mL^−1^ 2.4-D and 1 mg L^−1^ kinetin (control), CaO NPs (1 and 2 mg/L), CdCl_2_ (1 and 10 mM CdCl_2_) and combined treatments (1 mg/L CaO + 1 mM CdCl_2_, 1 mg/L CaO NPs + 10 mM CdCl_2_, 2 mg/L CaO NPs + 1 mM CdCl_2_ and 2 mg/L CaO NPs + 10 mM CdCl_2_). A total of 8 Petri dishes were used for each concentration in our study. After these treatments, the embryos were kept in a plant growth chamber with 16 h light/8 h darkness, 50% humidity at 25 °C and 1500 lx luminescence intensity for four weeks. The application groups and dosages were designed based on preliminary studies and previous reference studies [33,35].

### 2.5. RT-qPCR Analyses

The total RNAs from the callus tissues of two cultivars (*Yolboyu* and *Kirac*) were isolated using the SV Total RNA Isolation System (Promega, Madison, WI, USA) kit to determine gene (*Traes_5BL_9A790E8CF* and *Traes_6BL_986D595B9*) expression levels. RNA quality was evaluated, and RT-qPCR analysis was performed with primers *5BL* and *6BL* (Table 1) using 100 ng of the total RNA samples [36]. The cDNA was generated as a solution with a total volume of 5 µL consisting of 2 µL of 5U first chain buffer, 1 µL of 10 mM dNTPs, 1 µL of 0.1 mM DTT and 1 µL of reverse transcriptase (5 units/L). The following cycle conditions were used for the RT-qPCR reaction: 95 °C for 15 s, 55 °C for 30 s and 72 °C for 20 s for 40 cycles. Relative quantifications of gene expression data were established using the 2^−ΔΔCT^ or a comparative CT method [37]. Mtactin rRNA primers (Table 1) were used to standardize the results. PCR products were run in 1.2% (*w*/*v*) agarose electrophoresis using Ethidium bromide dye.

### 2.6. Determination of Proline and Soluble Sugar Contents

The method proposed by Rodriguez and Redman (2005) [38] was used to measure the proline content. In this method, 100 mg of callus tissue is homogenized in 5 mL of 3% aqueous sulfosalicylic acid and centrifuged at 4800 rpm for 15 min at 4 °C. In this study, 2 mL of extract were combined with 2 mL of acid-ninhydrin and 2 mL of glacial acetic acid in test tubes. After the samples were kept at 100 °C for 1 h, the reaction was finished by placing the samples in an ice bath. Then, 4 mL of toluene was used for extraction from this reaction mixture. The absorbance of the color reaction product was measured at 520 nm using toluene for a blank. Finally, proline concentration was determined with the aid of a calibration curve.

Homogenization of 100 mg of callus tissue was carried out using 5 mL of 2.5 N cold HCl. Afterward, centrifugation was carried out at 9000 rpm for 10 min. After centrifugation, the pellet was discarded and 2 mL of supernatant was transferred to a glass tube, and 2 mL of DNSA (3,5-dinitrosalicylic acid) solution was added. After this process, incubation at 90 °C was carried out in a water bath for 20 min. The mixture was kept in an ice bath until completely cooled. For each sample, 100 µL per well was added to a 96-well plate in triplicate. As a blank, 2 mL DNSA (3,5-dinitrosalicylic acid) and 2 mL 2.5 N HCl were added to 3 replications. In the final step, samples were measured in NanoDrop at a wavelength of 550 nm [39].

### 2.7. Determination of H_2_O_2_ and Malondialdehyde Content

To determine the H_2_O_2_ content, the method proposed by Sergiev et al., (2003) [40] was utilized. For this purpose, 0.2 g of callus tissue was homogenized with 5 mL of cold trichloroacetic acid (0.1%). The resulting solution was centrifuged at 12,000× *g* for 15 min at 4 °C. Then, 0.5 mL of 10 mM KH_2_PO_4_ (pH 7.0) and 1 mL of KI were added to 0.5 mL of the obtained supernatant. Absorbent values were measured and recorded at 390 nm [41].

The level of lipid peroxidation (LPO) was determined by assessing the malondialdehyde (MDA) level. MDA content was measured based on the method proposed by Heath and Packer (Heath and Packer 1968) [42]. Callus tissue (0.4 g) was homogenized in 4 mL of 5% TCA (Trichloroacetic acid). Subsequently, the homogenate was centrifuged at 13,000 rpm for 15 min. Then, 1 mL was withdrawn from the supernatant and then mixed with 1 mL of a 1% thiobarbituric acid (TBA) solution.

The tubes containing the resulting mixture were incubated in boiling water for 30 min and, subsequently, the mixture was placed in an ice bath and allowed to cool until it reached room temperature. Samples were centrifuged at 12,000 rpm for 5 min and supernatant aliquots were taken. The supernatants were subjected to centrifugation once again at 3500 rpm for 5 min, and the absorbance of the resulting supernatant was measured at 440, 532 and 600 nm using a microplate reader. The following calculations were performed based on the formula specified by Hodges et al. (1999) [43].

(1) MDA=nmolmL=[[A532−A600−A440−A600×0.0571]15700]×106

### 2.8. Energy Dispersive X-Ray Spectroscopy (SEM-EDX)

SEM-EDX analysis was used to determine the cell damage caused by CdCl_2_ and other treatments. A scanning electron microscope (SEM) (a Metek (Elmshorn, Germany), Apollo prime, active area 10 mm^2^, Microscope inspect S50) was used to determine the superficial structure of the synthesized CaO NPs. For this purpose, callus samples were incubated in a solution of 5% glutaraldehyde (pH 7.2, 0.1 M phosphate buffer) for 2 h at room temperature. Following the dehydration step using a graded ethanol series, water was removed from samples with a CPD (CO_2_ critical-point drying) system, sputter-coated with gold (Jeol JFC-1100 E ion-sputtering system), and samples were detected with a scanning electron microscope (HITACHI S-4700, Ibaraki, Japan) [33].

### 2.9. Statistical Analysis

In the experiment, two different wheat varieties were used for each analysis. A total of 54 Petri dishes, 3 for each concentration and wheat variety, were planted. Each Petri dish was accepted as an experimental unit. Three biological replicates were performed for each analysis. A two-way analysis of variance (ANOVA) was used for the data. For subsequent multiple comparisons, Duncan’s multiple range test was performed. All statistical analyses were performed with the Statistical Package for Social Sciences software (SPSS 26). In the statistical analysis, the significance levels were determined as *p* < 0.05.

## 3. Results

### 3.1. Callus Formation

**Figure 1:**
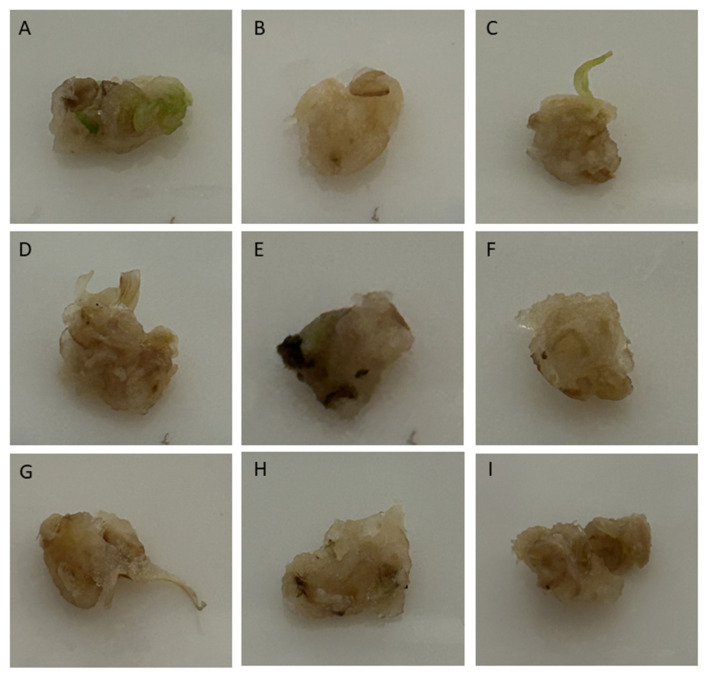
Callus of *Yolboyu* cultivar; the concentrations of the letters are as follows: (**A**): control; (**B**): 1 mg/L CaO NPs; (**C**): 2 mg/L CaO NPs; (**D**): 1 mM CdCl_2_; (**E**): 10 mM CdCl_2_; (**F**): 1 mM CdCl_2_ 1 mg/L CaO NPs; (**G**): 1 mM CdCl_2_ 2 mg/L CaO NPs; (**H**): 10 mM CdCl_2_ 1 mg/L CaO NPs; and (**I**): 10 mM CdCl_2_ 2 mg/L CaO NPs.

**Figure 2:**
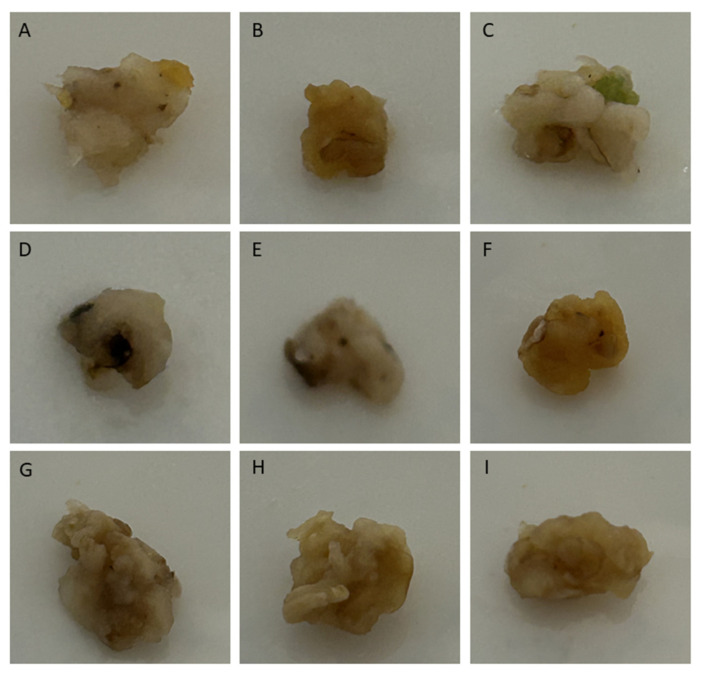
Callus of *Kirac* cultivar; the concentrations of the letters are as follows: (**A**): control; (**B**): 1 mg/L CaO NPs; (**C**): 2 mg/L CaO NPs; (**D**): 1 mM CdCl_2_; (**E**): 10 mM CdCl_2_; (**F**): 1 mM CdCl_2_ 1 mg/L CaO NPs; (**G**): 1 mM CdCl_2_ 2 mg/L CaO NPs; (**H**): 10 mM CdCl_2_ 1 mg/L CaO NPs; and (**I**): 10 mM CdCl_2_ 2 mg/L CaO NPs.

Only CaO NPs and low CdCl_2_ concentrations were shown to play a role in callus formation and development following Ca^2+^ uptake by the cells in wheat. In the *Yolboyu* cultivars, 10 mM of CdCl_2_ was found in the calluses, showing increased blackening and necrosis compared to the control and other treatments (Figure 1E), whereas CaO NPs and CaO NPs + CdCl_2_ showed synergistic effects and reduced callus necrosis (Figure 1F–I). In the images of the calluses in the *Kirac* cultivars, it was demonstrated that necrosis and Brown structures increased as the CdCl_2_ concentration increased (Figure 2). The 10 mM CdCl_2_/1 ppm CaO NPs and 1 mM CdCl_2_ treatments tolerated the stress effects the best (Figure 2F–I).

### 3.2. Quantitative Real-Time Analyses

**Figure 3:**
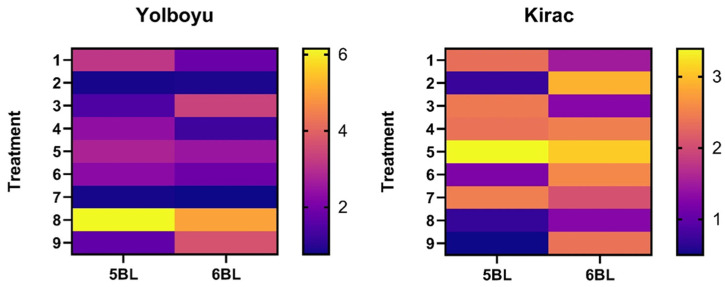
Heat map diagram of *5BL* and *6BL* gene expression levels for Cd stress genes analyzed by Real-Time Quantitative PCR. The heat map was drawn according to CT value. Columns and rows in the heat map represent treatment and genes, respectively. *Triticum dicoccum* Schrank ex Schübl cultivar names are displayed in the upper part of the heat map. Color scale indicates the fold changes in gene expression (determined using the heatmapgenerator5 tool). The numbers on the horizontal axis indicate the application of CaO NPs and CdCl_2_ at different concentrations and the control. The concentrations of the numbers are as follows: 1: control, 2: 1 mg/L CaO NPs, 3: 2 mg/L CaO NPs, 4: 1 mM CdCl_2,_ 5: 10 mM CdCl_2_, 6: 1 mM CdCl_2_ 1 mg/L CaO NPs, 7: 1 mM CdCl_2_ 2 mg/L CaO NPs, 8: 10 mM CdCl_2_ 1 mg/L CaO NPs and 9: 10 mM CdCl_2_ 2 mg/L CaO NPs.

The fold change in the *5BL* and *6BL* gene levels in the *Yolboyu* and *Kirac* varieties was calculated according to 2^−ΔΔCT^. The *Yolboyu 5BL* gene level was down-regulated in the control (3.143) and 10 mM CdCl_2_ 1 mg/mL CaO NPs (6.153), and the concentrations of the other NPs were up-regulated. The *6BL* gene was up-regulated compared to the control (1.816), 1 mg/mL CaO NPs (0.72), 1 mM CdCl_2_ (1.263) and 1 mM CdCl_2_ 2 mg/L CaO NPs (0.763), while the concentrations of the other NPs were down-regulated (Figure 3). The *Kirac 5BL* gene level was down-regulated in the control (2.326), 2 mg/L CaO NPs (2.426), 1 mM CdCl_2_ (2.37), 10 mM CdCl_2_ (3.39) and 1 mM CdCl_2_ 2 mg/L CaO NPs (2.47), while the other NPs were up-regulated. The *6BL* gene was up-regulated in the control (1.496), 2 mg/L CaO NPs (1.286) and 10 mM CdCl_2_ 1 mg/L CaO NPs (1.28), while the concentrations of other NPs were down-regulated (Figure 3).

### 3.3. Proline and Soluble Sugar Contents

**Figure 4:**
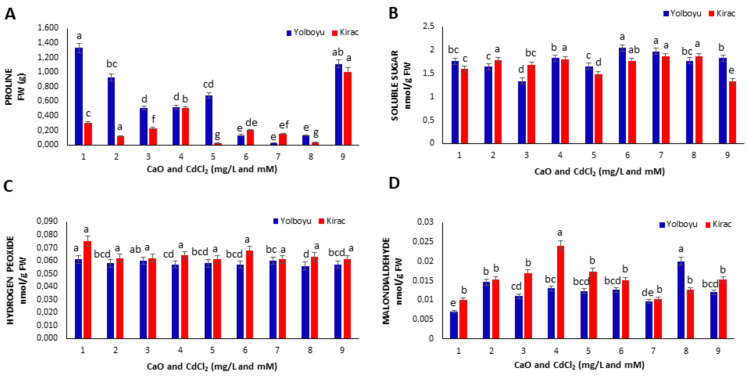
Physiological determination of Turkish Kavilca cultivars *Yolboyu* and *Kirac* at different CaO NP and CdCl_2_ concentrations compared with the control. (**A**) Proline content, (**B**) soluble sugar content, (**C**) hydrogen peroxide content, and (**D**) malondialdehyde content. a, b, c, d, e; Means in the same column with various superscript letters differ significantly (*p* ≤ 0.05). The numbers on the horizontal axis indicate the applications of CaO NPs and CdCl_2_ at different concentrations compared with the control. The concentrations of the numbers are as follows: 1: control, 2: 1 mg/L CaO NPs, 3: 2 mg/L CaO NPs, 4: 1 mM CdCl_2,_ 5: 10 mM CdCl_2_, 6: 1 mM CdCl_2_ 1 mg/L CaO NPs, 7: 1 mM CdCl_2_ 2 mg/L CaO NPs, 8: 10 mM CdCl_2_ 1 mg/L CaO NPs and 9: 10 mM CdCl_2_ 2 mg/L CaO NPs.

In this study, the proline and soluble sugar contents of the calluses were also evaluated under the same conditions (Figure 4A,B). We observed that the proline content of the *Yolboyu* cultivar was higher than that of the *Kirac* cultivar. On the other hand, two CaO NPs applications (1 and 2 mg/L) decreased the proline content in both cultivars at 1 mg/L (0.928 and 0.118 FW g) and 2 mg/L (0.508 and 0.228 FW g) compared to the control. Under CdCl_2_-only conditions, both CdCl_2_ applications (1 and 10 mM) decreased the proline content in *Yolboyu* compared to the control. However, under the same conditions, 1 mM CdCl_2_ increased the proline content in *Kirac* (0.501 FW g) while 2 mM CdCl_2_ decreased it (0.023 FW g). In the combined co-applications of CaO NPs and CdCl_2_, all CaO NP conditions except 10 mM CdCl_2_ + 2 mg/L CaO NPs decreased the proline content compared to their respective controls (Figure 4A). When the sugar contents of the same calluses were evaluated, 2 mg/L CaO NPs caused a significant (*p* < 0.05) decrease (1.335 nmol/g FW) especially in *Yolboyu*, while it did not cause a significant change in *Kirac*. CdCl_2_ applications alone generally did not significantly (*p* < 0.05) affect the sugar content in the calluses in both cultivars compared to the control under 1 mM conditions but decreased it under 10 mM conditions (1.658 and 1.481 nmol/g FW). When CaO NPs and CdCl_2_ were applied together to the *Yolboyu* variety, 1 mg/L CaO NP increased the sugar content under both 1 and 10 mM CdCl_2_ conditions, while 2 mg/L CaO NPs had no significant effect. In *Kirac*, 1 mg/L CaO NPs did not affect the sugar content under both CdCl_2_ conditions, while 2 mg/L CaO NPs caused an increase under 1 mM CdCl_2_ and a decrease under 2 mM CdCl_2_ conditions (Figure 4B).

### 3.4. H_2_O_2_ and MDA Contents

In cultivars, the effects of different treatments containing CaO NPs and CdCl_2_ on H_2_O_2_ levels and lipid peroxidation (LPO) were investigated (Figure 4C,D). When the H_2_O_2_ content was evaluated under CdCl_2_ stress in both wheat cultivars, only the application of CaO NPs decreased the H_2_O_2_ content in both cultivars, especially in *Kirac* (0.062 nmol/g FW) (Figure 4C). Similarly, both CdCl_2_ stress applications caused a decrease in H_2_O_2_ content compared to the controls. No significant change was observed in the H_2_O_2_ content in the groups where CaO NPs and CdCl_2_ were applied together compared to the controls (Figure 4C). The LPO level was evaluated by measuring MDA equivalents (Figure 4D). In vitro, both CaO NPs and CdCl_2_ applications significantly (*p* < 0.05) increased the MDA content in the calluses in both cultivars compared to their respective controls. These increases were found to be higher in the *Kirac* variety compared to the *Yolboyu* variety following the application of CdCl_2_. For example, applications of 1 and 10 mM CdCl_2_ caused increases in MDA content (0.044 and 0.0173 nmol/g FW). Conversely, when CdCl_2_ application was combined with CaO NP application, 1 and 2 mg/L CaO NPs decreased the MDA content in the calluses at both CdCl_2_ concentrations compared to controls in both varieties (Figure 4D).

### 3.5. Energy Dispersive X-Ray Spectroscopy (SEM-EDX)

**Figure 5:**
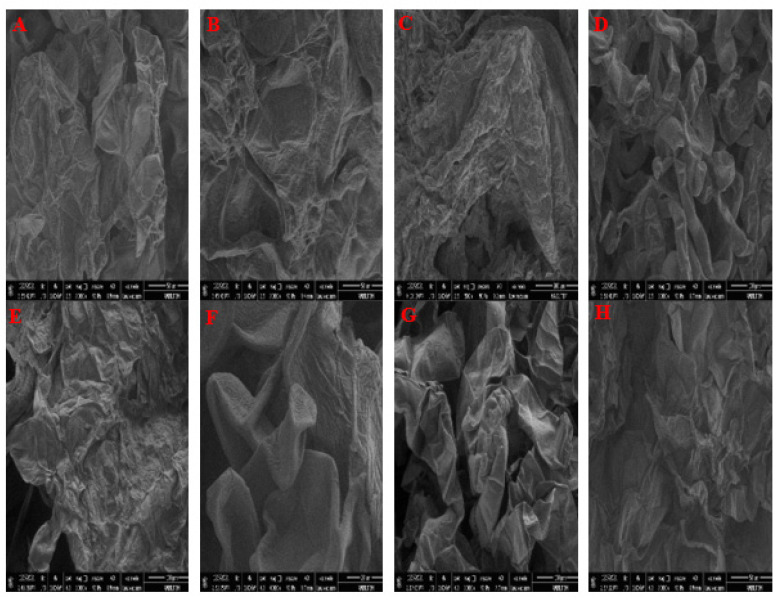
SEM images: (**A**): *Yolboyu* control, (**B**): 1 mM CdCl_2_, (**C**): 10 mM CdCl_2_, (**D**): 1 mM CdCl_2_ + 2 mg/L CaO NPs (**E**): *Kirac* control, (**F**): 1 mM CdCl_2_, (**G**): 10 mM CdCl_2_, (**H**): 1 mM CdCl_2_ + 2 mg/L CaO NPs.

SEM analysis was performed to confirm the structures of the callus tissues (Figure 5). Two wheat cultivars, *Yolboyu* and *Kirac*, were used to determine the stress response in the presence of CdCl_2_ at different concentrations using a scanning electron microscope. The analysis showed that the stress response of the callus tissues varied greatly across the various concentrations. Membranous structures were observed in both the control and CdCl_2_ treatments of the *Yolboyu* cultivars. However, the 1 mM CdCl_2_ + 2 mg/L CaO NPs treatment recovered structures of callus tissue in the *Yolboyu* cultivar. The mucilage structures of the callus in the control *Kirac* were converted to spherical and round clusters resembling SEM detections (Figure 5F,G). However, there was a continuous amorphous sphere or mucilage sphere on the callus’ surface in the *Kirac* treated with 1 mM CdCl_2_ + 2 mg/L CaO NPs.

## 4. Discussion

Previous research indicates that Cd can indeed have adverse impacts on plant metabolism [28,44,45,46]. However, studies suggested that Ca supplementation may mitigate Cd-induced stress in plants by promoting growth, regulating metal uptake and translocation, enhancing photosynthesis, reducing oxidative damage and modulating signal transduction pathways [9,47]. In addition, Ca^2+^ ions, which are involved in the protection of cell structure and stability, are an important factor in reducing the toxic effects of this heavy metal due to competition with Cd^2+^ in wheat roots [9]. Various metal nanoparticles like CaO NPs are used to reduce Cd toxicity in wheat [48,49]. For this reason, it is thought that CaO NPs may have critical importance in minimizing the unfavorable effects of Cd in wheat. Since Kavilca wheat is the ancestor of today’s modern bread wheat, it also carries some ancestral agricultural and economically valuable features. This is a considerable factor in this wheat species’ tolerance of various abiotic and biotic stress factors [50]. The CaO NPs treatment was able to contribute to the reduction in Cd uptake from the soil and the improvement of plant growth in barley [45] and mung bean (*Vigna radiata* L.) [46]. However, according to our knowledge, there is a significant lack of studies evaluating the effects of CaO nanoparticles on alleviating heavy metal stress, including Cd, under in vitro conditions. In addition, despite the abundance of studies illuminating the response mechanisms for Cd stress in modern wheat species such as *Triticum aestivum* and *T. durum* [15,51], there is insufficient information regarding the physiological, biochemical and molecular responses to heavy metal stress, including Cd, in ancient species like *T. dicoccum* (emmer or Kavilca wheat).

The callus cells exhibited an extensive brown color, which was more severe in the case of 10 mM CdCl_2_ (Figure 1 and Figure 2F). *Yolboyu* showed a recovery of Ca^2+^ NPs accumulation post heavy metal treatments. Similarly, callus necrosis intensively appeared on the *Kirac* cultivar in the case of the higher and lower CdCl_2_ treatments and the treatment with Ca^2+^ NPs. The severity of stress in the *Yolboyu* and *Kirac* cultivars treated with Ca^2+^ NPs was lower than those treated with sole CdCl_2_. Our results exhibited that the effects of Ca^2+^ NPs varied considerably depending on the genotype, stress levels and duration of treatments. A similar outcome of callus induction and development with CaO nanoparticle treatments under salt stress has also been reported [33]. The level of heavy metal tolerance is likely associated with the level of gene expression, which is related to changes in Ca^2+^ NP concentrations. These findings are in agreement with those published by Yazıcılar and Bezirganoglu et al., (2023) [35] in their study on *alfalfa* callus salinized at different NaCl dosages over a long period.

The gene units *Traes_5BL_9A790E8CF* and *Traes_6BL_986D595B9* play crucial roles, particularly in determining the adverse effects of cadmium heavy metals in winter wheat species. These genes, involved in metabolic and signal transduction pathways, alter their expression levels in response to stress conditions [13]. These genes are critically important as a reference, as they were previously used to measure the effect of cadmium Cd in cold-resistant winter wheat [14]. According to our findings, it can be argued that the CaO NPs obtained via the green synthesis play a complex role in regulating gene expression (*5BL* and *6BL*) in response to CdCl_2_ stress in the *T. dicoccum* cultivars (*Yolboyu* and *Kirac*) (Figure 3). Specifically, CaO NPs at concentrations of 1 and 2 mg/L increased the expression of the *5BL* gene in both wheat cultivars, irrespective of CdCl_2_ stress levels, suggesting a positive effect on the upregulation of the genes associated with the CdCl_2_ stress response of the wheat plants. However, the exposure to CdCl_2_ stress at both concentrations led to a significant decrease in the expression of the *5BL* gene in both wheat cultivars, with a more pronounced effect at higher CdCl_2_ concentrations. When combined with the CdCl_2_ stress, the CaO NPs generally mitigated the down-regulation of *5BL* gene expression caused by the CdCl_2_ stress. There were variations in response to the CaO NPs and the CdCl_2_ stress between the *Yolboyu* and *Kirac* cultivars. While both cultivars showed an up-regulation of *5BL* expression with 1 mg/L CaO NPs, the response to 2 mg/L CaO NPs and CdCl_2_ stress varied between cultivars. The effect of CaO NPs on the expression of the *6BL* gene was more variable compared to the *5BL* gene. While 1 mg/L CaO NPs generally led to an up-regulation of the *6BL* gene, the response to 2 mg/L CaO NPs was mixed, suggesting a dose-dependent effect of CaO NPs on gene expression. However, 1 mg/L CaO NPs partially mitigated the suppressive effects of CdCl_2_ stress on gene expression, indicating a potential protective role of CaO NPs (Figure 3). Xiao et al. (2019) [14] analyzed Illumina HiSeq4000 data from Cd-treated and untreated wheat roots to identify differentially expressed genes related to Cd stress. They also evaluated the differentially expressed genes responsible for plant hormone signal transduction (*Traes_4BS_6EC31E8D4*, *Traes_2BL_1AED96909*, *Traes_5BL_9A790E8CF*) and histidine metabolism (*Traes_6BL_986D595B9*) using q-PCR. They determined that some of these genes are up-regulated and some are down-regulated. The down-regulation of some of the expressed genes confirmed that Cd stress may not have a tangible effect during the initial period of plant development; however, it has also been reported that Cd harms plant metabolism. In a study in which Cd transporter gene expression was also examined, it was revealed that the addition of Ca to Cd-treated rice increased *OsNRAMP5* and *OsHMA2* gene expression in roots. Therefore, it was concluded that the addition of Ca up regulated the *OsNRAMP5* and *OsHMA2* genes [52]. However, a deeper understanding of the molecular mechanisms by which CaO nanoparticles regulate the expression of these genes is necessary.

When the proline content was evaluated in the same applications, the CaO NPs alone generally caused a decrease in proline content at both doses (Figure 4A). Studies demonstrating the effect of CaO NPs application on proline content, especially in calluses, are insufficient in the literature. The impact of the levels of Cd studied varied depending on the cultivars. In *Yolboyu*, both concentrations decreased the proline content, while in *Kirac*, the low concentration initially increased the proline content but then it decreased at higher doses. When CaO NPs and CdCl_2_ were applied together, the proline content generally decreased, but under certain conditions (e.g., 10 mM CdCl_2_ + 2 mg/L CaO NPs), an increase in the proline content was observed (Figure 4A). Increased proline accumulation is one of the defense mechanisms that plants develop against adverse environmental conditions such as drought, salinity, and heavy metal stresses. Free proline accumulation can vary depending on the developmental stage of the plant as well as environmental stimuli [53,54,55]. Although increases in proline content are suggested under heavy metal stress including Cd, the changes in proline content in plants under Cd stress are generally contradictory [55]. For instance, it has been proposed that Duckweed plants (*Lemna polyrrhiza* L.) and Ashwagandha (*Withania somnifera* Dunal) show an increased proline content at low cadmium levels, while higher doses decrease it [56,57]. Similarly, in another study conducted on pea plants under Cd stress, a significant decrease in proline concentration was observed in young leaves after 12 days of Cd application, supported by a decrease in the transcript level of the PsP5CS2 gene involved in proline synthesis [55]. It is known that Cd disrupts the uptake and movement of mineral nutrients in plants, particularly reducing nitrate uptake and inhibiting the activity of enzymes involved in N assimilation [57,58,59]. Therefore, we suggest that the decrease in free proline concentration in wheat calluses in response to CdCl_2_ might be due to a low nitrogen availability and proline ratio, which is expected to increase in response to CdCl_2_-induced stress, and is reversed by the curative effect of CaO nanoparticles.

Low concentrations of CdCl_2_ (1 mM) did not significantly affect the soluble sugar content in both cultivars, while high CdCl_2_ concentrations (10 mM) resulted in a decrease in sugar content. In addition, the co-application of CdCl_2_ and CaO NPs (1 mg/L) increased the sugar content. However, at high concentrations of CaO NPs (2 mg/L), there was no significant effect on sugar content (Figure 4B). The findings suggest that low concentrations of CdCl_2_ do not significantly alter soluble sugar content in both cultivars, implying a certain level of tolerance to low CdCl_2_ exposure. This is consistent with existing research indicating that plants have developed mechanisms to cope with low levels of heavy metal stress, possibly through the activation of antioxidant systems or metal detoxification pathways [57]. On the other hand, the decrease in sugar content observed at high CdCl_2_ concentrations aligns with previous studies demonstrating the detrimental effects of elevated Cd levels on plant metabolism, including the disruption of photosynthesis and inhibition of sugar synthesis pathways [60].

When the effects of both H_2_O_2_ and MDA levels on the physiological resistance of Kavilca wheat cultivars to CdCl_2_ were evaluated, the findings revealed a decrease in H_2_O_2_ values across all treatment groups compared to the control groups in both cultivars. (Figure 4C). As a result of the MDA analysis, it was observed that the damage to the Kavilca calluses formed in tissue culture increased with the applications compared to the control groups for both cultivars. While the damage increased with the application of CaO NPs to the *Yolboyu* cultivar, it was determined that it increased only with the application of low-dosage CdCl_2_ in the *Kirac* cultivar (Figure 4D). Reactive oxygen species such as superoxide, hydroxyl and hydrogen peroxide, create excessive ROS production under heavy metal stress, which is harmful to plants. These oxygen species affect membrane lipids and proteins through amino acid changes [57,60]. It has been reported that the peroxidation of cell lipids in wheat is significantly affected by Cd and Si exposure, and Cd toxicities significantly increase H_2_O_2_ and MDA in *T. aestivum* wheat [48]. In previous studies, lipid peroxidation and hydrogen peroxides were accepted as major criteria for the evaluation of oxidative stress damage [61]. The application of CaO NPs significantly reduced the levels of H_2_O_2_ and MDA in barley plants under arsenic [45] and Cd stress [62], while also increasing antioxidant enzyme activity and the levels of antioxidant molecules such as ascorbic acid and glutathione. When these results are considered alongside our study, they may indicate that CaO NPs play an important role in alleviating CdCl_2_ toxicity by regulating ROS metabolism not only in plants developed under in vivo conditions but also in the calluses developed from the two ancient wheat cultivars under in vitro conditions.

SEM analysis techniques can provide detailed information about stress responses, and identify the resistance of plant species to heavy metal stress. Microscopic analysis is commonly used to assess the impacts of stress on cells and has been found to be a fast and efficient method for their observation, confirming that it is beneficial in nanoparticle positioning in living cells [63]. The results show that the CaO NPs had the best-recovering structures when compared with the CdCl_2_ treatments alone in the SEM analysis (Figure 5). If these results are compared with those in the literature, it can be concluded that the same impacts have been detected [64]. We have detected a gradually enhanced physiological activity in somatic embryos at stages without NP treatment in *Triticale* callus tissues. This verifies that the application of NPs or any other development process has an impact on the recovery of physiological activity and thus enhances the stress response. The SEM images showed that the type of callus under each CdCl_2_ and CdCl_2_ + CaO NP treatment had a different callus structure compared to the control treatments. The membranous and fluffy character of the callus in the *Yolboyu* control changes to a partially wrinkled and compact structure, resembling the those detected under the 1 mM CdCl_2_ treatment. Conversely, there was a continuous wrinkled sphere, termed an extracellular matrix, on the callus samples from the 10 mM CdCl_2_ treatment. It was also observed that *Yolboyu* belonging to the CdCl_2_ + CaO NPs share mainly sectional cell structures and shapes (Figure 5A–D). In the *Kirac* genotype, among the three different CdCl_2_ and nanoparticle treatments, greatly varied callus images were observed compared to the control treatment (Figure 5E). The mucilage and the calluses in the *Kirac* control converted to pipe and torpedo shapes, resembling the 1 mM CdCl_2_ in the SEM detections. Conversely, there was serious damage and holes, and an extensive wrinkled sphere, on the 10 mM CdCl_2_. It was also observed that the CdCl_2_ + CaO NPs gave smooth and continuous amorphous structures to the callus samples (Figure 5F–H). Quite a different structure was observed after the CaO NPs treatment of the callus. In our images, we noted damage created by a high concentration of CdCl_2_, which altered the appearance of the callus structure as shown in Figure 5. Our results show that CaO NPs recover from heavy metal stress on SEM images. Similar results have been reported in Triticale [65] and in *Picea sitchensis* SE [66].

## 5. Conclusions

These findings point out the effects on the potential of CaO NPs in alleviating CdCl_2_ stress in ancient wheat cultivars, specifically *Yolboyu* and *Kirac*. The application of CaO NPs was shown to have a complex role in regulating gene expression, particularly the *5BL* and *6BL* genes associated with CdCl_2_ stress response pathways. While CdCl_2_ stress alone led to a significant decrease in gene expression, CaO NPs partially mitigated this effect, indicating a potential protective role. Additionally, the response to CaO NPs varied depending on the wheat cultivar and the concentration of NPs applied, suggesting genotype-specific responses to stress conditions. CaO NPs influenced physiological responses, including changes in proline content and soluble sugar levels. Proline showed varying trends in response to CdCl_2_ and CaO NPs. The co-application of low doses of CdCl_2_ and CaO NPs increased sugar content, indicating a potential role of CaO NPs in mitigating CdCl_2_-induced metabolic disturbances. CaO NPs also reduced H_2_O_2_ and malondialdehyde (MDA) levels. This reduction suggests that CaO NPs may regulate the metabolism of reactive oxygen species (ROS) and enhance antioxidant defenses, ultimately contributing to the alleviation of CdCl_2_ toxicity in wheat calluses. SEM analysis provided visual evidence that the CaO NPs played a potential role in maintaining cell integrity and structure by enabling the acquisition of smooth and more continuous structures in wheat callus cells under CdCl_2_ heavy metal stress and their protective effects. Overall, the findings presented in this study highlight the potential of CaO NPs as a promising approach for mitigating heavy metal toxicity in plants. Further research is warranted to elucidate the molecular mechanisms involved and to assess the efficacy of CaO NPs under field conditions.

Our research is currently in the period of somatic embryonic callus development. The regeneration of calluses will be accomplished by optimizing various combinations of auxins and cytokinins. In the near future, regenerated plants will soon be placed in soil, and morphological, physiological and genetic changes in the harmful effects of cadmium will be assessed.

## Figures and Tables

**Table 1:** Sequence and amplicon information for primers used in qRT-PCR.

Gene Id	Forward Sequence	Reverse Sequence
*Traes_5BL_9A790E8CF*	TTGGTGAGGTGACATGGGA	TGTTGCTGTCGTGGTCGTAG
*Traes_6BL_986D595B9*	CCACCATACTGCTAAACCCTC	GCGTCGTTGAATGTGATGC
*Mtactin 18S rRNA*	TGACGGAGAATTAGGGTTCG	CCTCCAATGGATCCTCGTTA

## Data Availability

Data are contained within the article.
